# News

**DOI:** 10.1155/S1463924602000056

**Published:** 2002


Journal of Automated Methods & Management in Chemistry
Vol. 24, No. 1 (January–February 2002) pp. 27–28

Taylor & Francis

Taylor & Francis Group

### News

### Pharmaceutical Resources Inc. selects VelQuest solution to enable paperless laboratory operations in their quality & development organizations

*Par Pharmaceuticals, a division of PRI, expects the Paperless Laboratory to help expedite the launch of new Generic Pharmaceutical Products.*

*VelQuest Corporation, Hopkinton, MA—15 January 2002*: VelQuest, the innovator of Electronic Process Management and Compliance Solution (ePMC$^{TM}$), and Pharmaceutical Resources Inc. (PRI) (NYSE PRX), the fastest growing stock in 2001 according to the NY Times and leading US pharmaceutical company, announced today that PRI has committed to implementing the VelQuest's Compliance Electronic Notebook platform as a key part of their overall quality, compliance and data architecture plan.

'A critical challenge faced by generic pharmaceutical companies is to be first to market with generic drugs', said Robert Femia, Vice President of Scientific and Regulatory Affairs at Par Pharmaceuticals. 'Leveraging our operations with VelQuest's ePMC Solution will allow us to be more efficient and help to expedite our efforts to bring drugs to market'.

To date, the typical process for preparing ANDA/NDA submissions is paper-based and involves hours of reviewing, retrieving and interpreting written data. Par Pharmaceuticals has taken the initiative to implement an electronic process management and compliance platform using VelQuest's ePMC paperless solution. VelQuest's ePMC solution with electronic notebooks will be deployed in Par Pharmaceutical's quality & development operations, providing an electronic paperless quality solution for their data and documentation.

***Pharmaceutical Resources has taken other initiatives in computer-based efficiency enhancements through its recent acquisition of a controlling interest in High Rapids Technologies, a software firm that specializes in the implementation of artificial-intelligence-based applications within specific markets, such as the pharmaceutical industry. PRI's selection of VelQuest's Compliance Electronic Notebook platform was identified as a 'best-in-class' product to complement parallel advances in operational systems being made through the High Rapids technology.***

'We are very excited to complement PRI's investment in High Rapids technology within their operations', said Ken Rapp, President and CEO of VelQuest Corporation. 'Generic Pharmaceuticals are very important to consumers and PRI continues to enjoy a leadership position helping consumers with unique Generic alternatives in the pharmaceutical market'.

VelQuest's ePMC platform is an innovative solution to the fundamental problem that pharmaceutical laboratory records are frequently paper-based and laboratory notebook documentation and data-auditing-related activities can consume up to 70% of valuable human resource. VelQuest's solution interacts with scientists in real time and allows the capture of data directly from human observations, electronic devices such as balances and pH meters, and PC-controlled instruments. VelQuest's solution was designed with compliance (cGMP, GLP, 21 CFR Part 11) as the core functionality. The company provides a comprehensive solution including technology and services that enable pharmaceutical companies to increase productivity and create an electronic compliance platform.

Currently, VelQuest is deploying their ePMC solution at several global pharmaceutical companies. The company plans to continue to develop additional productivity and compliance-oriented technology for the pharmaceutical laboratory environment.

### *About VelQuest*

VelQuest Corporation, headquartered in Hopkinton, MA, the innovator of an electronic compliance platform enabling the paperless laboratory using electronic notebooks, develops quality and compliance technology for pharmaceutical development and quality operations. VelQuest's ePMC solution provides a fully validated, paperless approach to data acquisition, documentation, storage and retrieval for use in new-product submissions, product releases, stability testing, quality assurance and management analysis. More information can be found at www.velquest.com.

### *About Par Pharmaceuticals*

Par Pharmaceutical Inc., located in Spring Valley, New York, manufactures and distributes over 100 products representing various dosage strengths of almost 50 drugs. Par Pharmaceutical is dedicated to developing, manufacturing and marketing the highest-quality generic pharmaceutical products for value-conscious healthcare consumers. Our strength is the integration of leading pharmaceutical technologies with quality and cost-efficient manufacturing. For press releases and other Company information, visit the website at http://www.parpharm.com.

Certain statements herein constitute 'forward-looking statements' within the meaning of the Private Securities Litigation Reform Act of 1995, including those concerning management's expectations with respect to future events or future financial performance. Any such statements that refer to either VelQuest's or PRI's anticipated future results, product performance, or other non-historical facts are forward-looking and reflect PRI's current perspective of existing trends and information. These statements involve risks and uncertainties that cannot be predicted or quantified; consequently, actual results may differ materially from those expressed or implied by such forward looking statements.

*For further information, please contact Ed Halpin, Tel: + 1(508) 497 3629, e-mail: edh@velquest.com*

